# Exploring Tools for Designing Dysphagia-Friendly Foods: A Review

**DOI:** 10.3390/foods10061334

**Published:** 2021-06-10

**Authors:** Larisa Giura, Leyre Urtasun, Amanda Belarra, Diana Ansorena, Icíar Astiasarán

**Affiliations:** 1 Department of Nutrition, Food Science and Physiology, Faculty of Pharmacy and Nutrition, Instituto de Investigación Sanitaria de Navarra, Universidad de Navarra, IDISNA, C/Irunlarrea s/n, 31008 Pamplona, Spain; lgiura@alumni.unav.es (L.G.); iastiasa@unav.es (I.A.); 2 National Centre for Food Technology and Safety (CNTA)—Technology and Knowledge for Food Sector Competitiveness, Navarre, Crta-Na 134-km 53, 31570 San Adrian, Spainlurtasun@cnta.es (L.U.); abelarra@cnta.es (A.B.)

**Keywords:** dysphagia, food texture, rheology, thickeners, swallowing, viscosity, viscoelasticity, 3D printing

## Abstract

Dysphagia is a medical condition that affects normal swallowing. To prevent the risk of aspiration or choking, thickened fluids and texture-modified foods have been used for dysphagia management with the goal of slowing down the flow of liquids and protecting the airway. This article summarizes the available information about the rheological and textural parameters, the characterization of the most-used thickeners and the application of alternative texture modification technologies that are crucial to developing safe dishes for people who suffer from swallowing difficulties. Regarding rheological and textural measurements, fundamental and empirical methods are described.

## 1. Introduction

Dysphagia is a symptom of a swallowing disorder that causes difficulty or the inability to form or move the food bolus safely from the mouth to the stomach. It is estimated to affect approximately 8% of the world’s population (https://iddsi.org/, accessed on 9 June 2021), with a higher prevalence among the elderly as compared to other ages. This condition is also commonly caused by neurological or structural disorders such as stroke, Parkinson’s disease, dementia, head and neck cancer, multiple sclerosis, spinal cord injury or by clinical practices [1,2,3,4]. As clinical practices, early intubation and mechanical ventilation are commonly needed to manage Acute Respiratory Distress Syndrome in COVID-19 patients, and attention should be paid to these patients in relation to potential dysphagia problems [1]. Swallowing dysfunction might cause reduced oral intake, leading to weight loss, malnutrition and dehydration [5]. Moreover, people with dysphagia are commonly affected by choking and aspiration when eating or drinking. These situations could possibly lead to aspiration pneumonia, resulting in extended hospitalization, weakness, illness, anxiety and even lower survival rates [6,7].

As a result, it is critical for these patients to ingest texture-modified foods (TMF) to minimize chewing impairments or fatigue and thickened fluids with the goal of slowing down the swallowing process, making it safer and more efficient [8,9]. Nevertheless, although texture modification is one of the most popular dysphagia intervention techniques, the definitions of thickened drinks and texture-modified foods differ worldwide [10]. Different classifications have been used so far for that purpose. One of the first ones was that published in 2002 by the American Dietetic Association, the National Dysphagia Diet (NDD), that proposed four categories for classifying the consistency of fluids. Later, the International Dysphagia Diet Standardization Initiative (IDDSI) released, in 2016, an international framework to standardize terms and definitions to classify not only drinks, but also foods used in dysphagia management by eight levels [11].

Regarding the design of optimal food products for the treatment of dysphagia, rheological and textural properties, including shear viscosity, viscoelasticity, yield stress, extensional viscosity, hardness, cohesiveness, adhesiveness and more recently, tribology are very important parameters to be taken into consideration [12,13,14,15,16,17].

The use of thickeners for modulating these properties is widely extended, as well as the application of some processing conditions able to affect the rheological properties of foods. In this sense, the use of technologies such as high-pressure processing (HPP) or 3D food printing have been applied to modify the texture and sensory characteristics of dysphagia foods and preserve their safety and nutritional value [18,19,20,21].

Therefore, the purpose of this article is to summarize the available information about the current trends used for dysphagia food management, including the characterization of rheological and textural properties of optimal products designed for dysphagia, the description of the thickeners used in their elaboration and the review of applied industrial technologies for food texture modification.

## 2. Textural Characterization of Dysphagia Food Products

### 2.1. Empirical Methods of Measuring Rheological Properties

Rheology plays an important role in understanding the texture in food’s oral processing and swallowing. Modifying liquid and food viscosity influences their flow properties, which is important for reducing the risk of aspiration and optimizing the swallowing ability of patients with dysphagia. These textural and rheological properties of foods can be quantified by empirical, imitative and fundamental rheology measurements. 

Regarding empirical methods, in hospitals and industry, several tools have been used, such as the line spread test, the Bostwick consistometer method or the previously mentioned IDDSI method, because of their simplicity and accessibility as compared to the use of equipment such as a viscometer, tribometer or rheometer.

The line spread test (LST) is a practical and rapid analytical test used to measure the flow distance (in cm) that a thickened fluid runs across a flat surface. The system consists of a white sheet of paper with concentric circles drawn at 1 cm intervals to represent the fluid spread distance, and a cylindrical tube of 5 cm diameter and 3.5 cm high that is placed in the center of the circles. Once the thickened sample is filled, the cylinder is lifted and the sample is allowed to flow on the flat surface. After 1 min, the measurements in centimeters along the four lines that divide the quadrants are registered. Kim et al. [22] suggested that the LST is a reliable and simple method for evaluating the correct and desirable viscosity for a dysphagia diet, and can be used to differentiate between nectar-like, honey-like and pudding-like consistencies of thickened fluids. More recently, Merino et al. [23] employed a new version of the LST to assess the adequate consistency of blended dishes for cerebral palsy dysphagic people in order to select the most appropriate texturizing agents for that purpose.

The Bostwick consistometer is another simple tool, accessible and commonly used in the food industry to measure the thickness consistency and flow rate of the material. The device is composed of one compartment of 5 × 5 × 3.8 cm high and one horizontal trough 5 cm wide, 24 cm long and 2.5 cm high, which has 0.5 cm gradations along the bottom. This method involves the fluid flow, from the compartment in which the liquid is loaded previously, due to gravity as soon the gate of the compartment is opened. Consistency is determined by the distance traveled by the liquid over 30 s. In a comparative study about the consistency of different thickened fluids measured by simple tools, it was suggested that the Bostwick consistometer and LST are potential methods for differentiating consistency levels and underlined the need for guidelines for more objective measurement standards [24].

The IDDSI Methods (International Dysphagia Diet Standardization Initiative) provides a common terminology to describe drink thickness by levels from 0 to 4: Thin, Slightly, Mildly and Moderately thick; and food textures by levels from 3 to 7: Liquidized (moderately thick), Pureed (extremely thick), Minced and moist, Soft and bite-sized, Easy to chew and Regular. By means of text labels, numbers and color codes, the different levels are identified. One of the advantages of this method is that it uses basic tools. For example, dysphagia drinks (levels 0–4) are measured by the gravity flow test in which a syringe with a measured length of 61.5 mm from the zero line to the 10 mL line is used to quantify the liquid’s flow category (sample remaining from 10 mL after 10 s of allowed flow is recorded) [10,11].

For dysphagia foods (levels 3–7), the IDDSI employs simple tools such as a spoon, a standard metal fork, chopsticks or the fingers. A spoon tilt test is recommended for determining cohesiveness and adhesiveness. When rotated or turned sideways, the food should keep its shape on the spoon, fall easily and leave little residue. These characteristics result in a moist, cohesive bolus that is not sticky or adhesive. To measure the hardness of the sample, a practical test using a fork is recommended. The IDDSI method contains no measuring equipment and, as a result, no numerical criteria for textural and rheological attributes; thus, it can be used by caregivers, speech therapists and patients [10].

In any case, this technique is applicable for frontline applications, although it has been suggested that *it* is not the most appropriate one for quality control purposes in laboratories at both the manufacturing site or at the research level [12].

Côté et al. [25], in a review over the evidence for a potential validation of the suggested IDDSI descriptors and metrological properties of the IDDSI testing methods in the geriatric context, concluded that future work must provide objective, quantitative, validated and reliable measurement tools to food and pharmaceutical industries in order to develop efficient food-treatment strategies.

In this sense, it has been reported that food bolus properties seem to be a key feature that should be instrumentally measured [26]. These authors suggested that a viscometer is an appropriate instrument to measure the viscosity of a liquid; a texture analyzer would be optimal for the evaluation of the texture of solid or gel-type food and the viscoelasticity of a fluid can be observed by employing a rheometer.

### 2.2. Fundamental Methods of Measuring the Rheological Properties of Dysphagia Foods

In the last few years, various studies have demonstrated that for optimal modification of the food texture it is necessary to know not only the viscosity, but also other important parameters such as bolus viscoelasticity, yield stress, propulsion pressure, extensional viscosity, mechanical properties and the degree of lubrication of dysphagia foods [10,13,15,17,27,28,29,30]. Therefore, various fundamental testing methods can be performed to obtain all these parameters. 

#### 2.2.1. Rotational Shear Rheometer

The most common fundamental method to measure the rheological properties of thickened foods is by performing Steady Flow Sweep tests, which provide information about the viscosity of the sample, and Dynamic Oscillatory tests, which provide data about the viscoelastic behavior of the sample. These measurements are obtained with a rotational strain-controlled rheometer by using concentric cylinders, stainless-steel parallel plate–plate or cone–plate geometries. The geometry chosen is determined by the viscosity of the thickened fluid.

##### Steady Flow Sweep Tests

Most of the available information regarding the fundamental rheological properties of dysphagia-thickened liquids is mainly focused on shear viscosity. Viscosity is the resistance of a fluid to flow under an applied force and is typically measured by performing Steady Flow Sweep tests, where the fluids are analyzed at a steady applied shear rate and the resulting shear stress is measured. The apparent viscosity η (Pa.s) is defined by the shear stress σ (Pa) applied to this fluid divided by the shear rate $\dot{\gamma \;}$(s^−1^) Equation (1).

(1) $$\eta =\frac{\sigma }{\dot{\gamma }}$$

In the field of dysphagia, the viscosity of food products is commonly measured with a rheometer at 25 °C at a shear rate of 50 s^−1^, according to the National Dysphagia Diet Task Force and American Dietetic Association, 2002. At this shear rate value, the viscosity of the four NDD categories are defined as thin (0–50 cp), nectar-like (51–350 cp), honey-like (351–1750 cp) and pudding-like (>1750 cp).

In 2018, Ong et al. [31] pointed out that, even though a shear rate above 50 s^−1^ is recommended to represent the pharyngeal shear rate during swallowing, this will likely be influenced by sample viscosity, and it is unlikely that a single shear rate can be applicable for products of all viscosities. Flow rates during swallowing are also likely to be different depending on the age of the person and his or her level of dysphagia [10].

Given the current lack of information about the shear rate at which the bolus is subjected during swallowing, Gallegos et al. [32] suggested that it would be important to characterize the viscosity of boluses over a wide interval of shear rates.

Considering this approach, it has been observed that thickened foods used in dysphagia management can be described as shear thinning; namely, lower viscosities are observed at higher shear rates. The Power Law Model (Equation (2)) has been applied to report the data of shear thinning of dysphagia food products. In this model, *η* indicates the apparent viscosity (Pa.s), *k* is the consistency index (Pa.s^n^) which describes the overall range of viscosities across the part of the flow curve that is being modeled, $\dot{\gamma \;}$ is the shear rate (s^−1^) and n defines the flow behavior index (dimensionless).

(2) $$\eta =k{\dot{\gamma }}^{n-1}$$

Both *n* and *k* values are obtained by running a shear rate sweep test (plotting apparent viscosity versus shear rate).

The flow behavior index indicates how the viscosity changes depending on the shear rate. If the value of *n* is 1, the fluid is a Newtonian fluid (the viscosity does not modify when increasing the shear rate); an *n* value below 1 indicates a shear-thinning behavior (a decrease of viscosity) and an *n* value above 1 is shear-thickening (an increase of viscosity).

Several studies have used the Power Law model to compare the consistency and the degree of shear-thinning of different thickened foods for dysphagia diets [26,33,34,35].

##### Dynamic Oscillatory Tests

At a selected temperature and shear rate, TMF with different thickening agents may present similar apparent viscosity, but can exhibit different flow behaviors or viscoelastic properties. These parameters are particularly important in predicting the stability of a fluid or determining the integrity of the bolus in order to develop safe dishes for patients with dysphagia.

Viscoelasticity is a feature of a material that exhibits both viscous and elastic behavior. When measuring a material's viscoelastic behavior, the Linear Viscoelastic Region (LVR) is determined first for characterizing the sample without affecting the food structure during measurement. The LVR of a material is determined by performing an Oscillation amplitude sweep test with a strain ranging from 0.01% to 1000%.

The material stiffness and rigidity can be defined by the complex modulus G* (Pa) which is composed of two parts: a storage modulus (G′), which indicates the solid-like or elastic behavior and a loss modulus (G′′), which indicates the liquid-like or viscous behavior.

The phase angle (δ) and the loss tangent (tan δ) are also often used to characterize thickened fluids and express the relative aspects of the viscous and elastic components. The phase angle represents the phase difference between the applied strain and the response measured in the material. In a purely elastic behavior, the phase angle is δ = 0° (Hookean Solid), while in a purely viscous behavior, the phase angle is δ = 90° (Newtonian liquid). A phase angle between 0° and 90° denote a viscoelastic response. The loss tangent (tan δ) describes the viscoelastic balance of the material (Equation (3)).

(3) $$\tan\;\delta =\frac{G\prime \prime }{G\prime }$$

Therefore, strong gels have tan δ values below 0.1, dilute solutions have tan δ values above 1, and weak gels have tan δ values between 0.1 and 1. According to Sharma et al. [36], a higher tan δ value and, as a result, a higher viscous component (G′′) can make food boluses easier to swallow.

This parameter (tan δ) has been extensively used in the literature to describe the viscoelastic properties of dysphagia foods [20,35,36,37,38,39].

Suebsaen et al. [37] characterized banana dessert gels for the elderly with dysphagia. They found a high positive correlation of tan δ with the security scores for swallowing and hardness. Interestingly, the elderly persons in the sample preferred the banana gel with soft texture (high tan δ). Soft gels are considered safe and can be easily made palatable. Consequently, they have been suggested to be used in hospitals and institutions that take care of dysphagia-affected persons [40].

In rheology, G′, G′′ and the loss tangent (tan δ) are usually characterized by an Oscillation Frequency Sweep test within the LVR and thus can help to predict the mechanical strength of a food product over time. 

The yield stress is another studied rheological parameter in dysphagia food management that refers to the minimum stress needed to cause a fluid to flow. It also provides data about the strength of the internal structure of the network structure in concentrated suspensions [41]. This parameter can be measured by performing shear stress/shear rate flow curves and then fitting a rheological model such as Bingham, Casson, Herschel–Bulkley or by performing an Oscillation Amplitude Sweep test where the crossover point is the yield stress of the sample.

Ross et al. [42], in a study that aimed to find correlations between shear rheology and the textural properties of thickened fluids elaborated with hydrocolloids, suggested that those which are highly shear-thinning and have yield stress present better flow properties to guarantee the safety and the therapeutic efficiency in patients with dysphagia.

Another study by Abu Zarim et al. [43] about the effect of different concentrations of thickeners in texture-modified chicken rendang for individuals with dysphagia reported that the addition of 30% carboxymethyl cellulose gum showed the highest yield stress value as a consequence of a stronger interaction between particles, making it suitable for the development of dysphagia foods. Such interaction is essential for the formation of a cohesive bolus, which minimizes the disintegration of foods and thus the risk of aspiration.

#### 2.2.2. Extensional Rheometer

In the swallowing process, the extensional viscosity is also significant and it is strongly correlated to the cohesiveness of thickened fluids. 

Hadde and Chen. [14] suggested that high extensional viscosity reduces the risk of breakage of the bolus due to reduced elongation while swallowing because of its effect on the cohesiveness of the fluid. The extensional viscosity of a fluid can be measured by an extensional rheometer.

Scientific literature has been mainly focused so far on the shear deformation of thickened fluids. However, there are still few studies dealing with the extensional deformation of thickened fluids [14,15,30].

#### 2.2.3. Tribometer

Other important parameters in oral food processing include tribological aspects related to the interactions between the mouth tissues and the food surface, which could be relevant to the swallowing process, and consequently, to patients with dysphagia [16,44]. One of these parameters is slipperiness, or lubrication of the samples, which describes how easy the food slides over the tongue [31]. In research laboratories, tribometers are commonly used to measure these properties.

Vieira et al. [13] in a study about rheology and soft tribology of different dysphagia-oriented thickened dispersions, reported that gum-based formulations have better lubrication capacities than starch-based solutions, and therefore provide a better swallowing process by avoiding the unpleasant stickiness feeling. In the last decade, tribology science has experienced a growing interest and it is expected that in the coming years, it will provide valuable methods for the good characterization of foods.

#### 2.2.4. Texture Analyzer

The mechanical properties of TMF are important in the swallowing process. Regarding food texture, the literature highlighted hardness, cohesiveness and adhesiveness as being relevant parameters both for physiological behaviors and bolus flow patterns [10,45]. These parameters are generally associated with the bolus preparation in the oral stage and can be measured in a laboratory with a texture analyzer. Hardness (N) is defined as the maximum force that is required to compress food between the tongue and palate to a given deformation or to penetration; adhesiveness is defined as the work required to remove food that adheres to the mouth (generally the palate) during the normal swallowing process; cohesiveness means the strength of internal bonds making up the body of the food, and gumminess (N) described the energy needed to transform a semi-solid food into a bolus able to be swallowed [46,47].

The Japanese dysphagia-modified diet defines “easy to swallow foods” as those whose texture can be defined by the three following criteria: hardness under 15,000 N/m^2^, adhesiveness under 1000 J/m^2^ and cohesiveness between 0.2 and 0.9 [48]. TPA (texture profile analysis) and back-extrusion tests are mainly used to determine these properties.

Park et al. [47], studying the effect of texture properties of semi-solid food on the effort of the pharyngeal swallow in older adults, found that adhesiveness was significantly correlated with both sensory tests (difficulty to swallow and sense of residue), with the lowest values (in the same viscosity category) being those that make it easier to swallow.

Xie et al. [49] used a back-extrusion test to determine the firmness and consistency of a soft-fish paste in order to validate, by means of instrumental determinations, its classification in level 4 (pureed and extremely thick) of the IDDSI. An overview of rheological techniques that have been used in the last few years and their applicability to characterize dysphagia foods are shown in Table 1.

## 3. Thickeners

Hydrocolloids are commonly used in dysphagia management to improve the consistency and cohesiveness of foods and make them safe to swallow [45,54,55,56,57]. Most available dysphagia products are ready-to-use commercial thickeners that can be added to various types of food matrices in order to increase bolus viscosity and decrease aspiration risk. Table 2 describes some of the most relevant characteristics associated with usually employed thickeners. In most cases, thickeners are used at concentrations needed to obtain viscosity values within the ranges for honey (351–1750 cp) or pudding (>1750 cp).

Among the most used ones, modified starches and gums are highlighted. Starch-based thickeners such as modified corn starch, tapioca starch, potato starch or sago starch have been used due to their simple preparation, good availability worldwide and low cost. Chemically, modified starch is a carbohydrate polymer composed of amylose and amylopectin molecules which increase the viscosity of a fluid by absorbing the water of the dispersing media and swell [62].

Despite the fact that starch-based thickeners are still widely used in dysphagia products, they present some limitations in taste, viscosity, stability and solubility. Thickened foods altered by adding modified starch are characterized by their starchy taste, grainy texture and cloudy appearance. Their viscosity increases over time and they show a higher prevalence of oropharyngeal residue as compared to other thickeners. Moreover, they can suffer hydrolysis by contact with amylase in the saliva which may cause a thinning effect over time, which can lead to potential safety problems, given that dysphagia patients usually take more time to eat and drink than normal [55,62,63].

In order to counteract these inconveniences, other alternatives such as gum-based thickeners have been explored. Gums are hydrocolloidal gels-polysaccharides that exhibit good binding properties with water and other materials, increasing the viscosity of fluids by causing meshes of entanglement that water becomes lodged in [64,65].

Thickened foods modified by adding gum-based thickeners present a soft uniform texture, have better taste, are stable over time, are not affected by amylase, are stable over a large range of temperatures and pH and have the ability to form thicker fluids and to provide lower oropharyngeal residues at lower concentrations than starches [55,59,62].

In particular, xanthan gum has been investigated extensively for formulating dysphagia foods; [20,21,26,33,42,54,57,59,62,66]. Xanthan gum is a microbial polysaccharide produced by the bacterium *Xanthomonas campestris* and is commonly used in dysphagia foods due to its high viscosity at low concentrations, its temperature changes stability and ionic strength in comparison with other thickening agents [65,67,68].

In 2014, Rofes et al. [59], in a study about the effects of a xanthan gum-based thickener on the swallowing function of patients with dysphagia, reported that these thickeners showed higher protection on swallow process as compared to starch-based ones. Consequently, they suggest them to be a new generation of thickeners with better therapeutic value. 

A recent study by Ross et al. [42] comparing starch, carboxymethylcellulose and xanthan gum has concluded that the last one showed the most suitable oral cohesiveness related to the perceived oral propulsion effort, stickiness and oral residue. 

Besides xanthan gum, other thickeners such as pectin, carboxymethyl cellulose, tara gum, konjac [35,43], guar gum [20,31], gellan gum [36,61], agar, carrageenan and gelatin [37,69] have been investigated in order to achieve safe swallowing. 

Gellan gum has been studied as a new potential member in the dysphagia thickener family. In 2019, Torres et al. [61] compared rheological, zeta-potential and tribological properties of gellan gum against commercial starch-based thickeners and xanthan gum. The apparent viscosity unaffected by α-amylase, and the good lubrication profile of gellan gum highlighted the promise of it as a potential dysphagia thickener. Nevertheless, the authors suggested that more studies are needed to warrant its application in dysphagia management.

Guar gum is a useful material to study because of its high propensity to form hydrogen bonds in water, making it a novel thickener and stabilizer [70]. In 2020, Dick et al. [20], in a study about the feasibility of various hydrocolloids incorporated in a pork paste for dysphagia people, reported that at high shear rates, when a shear-thinning behavior is observed due to a decreased viscosity of the gums in a solution, it is noticed that xanthan gum and guar gum provide more viscoelasticity and stability to the pastes as compared to the sample control, proposing their suitability for 3D-printing processing by extrusion.

Recently, flaxseed gum was suggested to be a good alternative thickener due to its rheological properties at neutral and acidic pH. Flaxseed gum is a soluble polysaccharide extracted from *Linum usitatissimum* and is composed of polysaccharide fractions, including fibers that, besides their technological role, have been associated to several health benefits such as an improvement in intestinal tract transit, in postprandial glycemia and in weight control, among others [60,71].

Carboxymethylcellulose is also used in dysphagia foods because of its capacity to form soft gels. A recent paper by Talens et al. [35] on the flow, viscoelastic and masticatory properties of tailor-made thickened pea cream for people with swallowing difficulties found that using carboxymethyl cellulose, tara gum and konjac gum to improve the texture of thickened cream provide a greater viscous component that resulted in a bolus that was easier to swallow. 

In a recent paper, Wei et al. [39] studied the efficacy and applicability of carboxymethylated curdlan and konjac glucomannan and their mixture in water and in model nutrition emulsions in order to design new dysphagia products. Rheological characterization was compared with those obtained with xanthan gum. Carboxymethylated curdlan showed a unique viscosity-enhancing ability, presenting promising feasibility as a novel dysphagia-oriented thickener. On the other hand, konjac glucomannan showed a good thickening ability in water, but a significant viscosity-lowering phenomenon occurred in the model emulsion containing maltodextrin, resulting in a remarkably different thickening behavior from that of xanthan gum. However, the authors suggested that given the beneficial properties of konjac glucomannan as dietary fibers, maltodextrin's antagonistic impact on its viscosity offers an opportunity to use more dietary fibers in dysphagia diet formulas.

All these results showed that a high variety of dishes, including pureed foods, milk formulas, fruit juices, vegetable creams and meat/fish patties have been developed in order to obtain the appropriate textural properties (analyzed by different analytical methods) and using different thickeners (Table 1 and Table 2).

Moreover, in general, thickeners are exposed to a variety of conditions that may alter the proper preparation of modified food. When developing foods for people suffering dysphagia, important preparation variables such as previous standing time, the pH of base fluid, amounts of thickener to achieve the desired viscosity, serving temperature, dispersing media and sensory acceptance must also be taken into consideration [28,72,73,74,75,76,77].

MacHado et al. [77] investigated seven commercial thickeners in regard to their composition, applied terminology, instructions needed for their preparation and recommended amount for obtaining level two viscosity and nutritional information. The authors suggested that a lack of details in relation to the time of preparation, base liquid and temperature was noticed, and no correlation between viscosity classifications provided by NDD and IDDSI was found.

## 4. Texture-Modification Technologies

### 4.1. HPP

HPP is a non-thermal preservation technology in which foods are subjected to high pressures (from 100 to 900 MPa) in order to destroy pathogenic microorganisms and enzymes, extending the shelf-life of foods. As no heat is applied, the sensory and nutritional properties of foods are interestingly preserved [78]. Foods are sealed in their packages, introduced into a chamber and water is used to transmit the pressure. This pressure affects non-covalent bonds of molecules and consequently, is able to modify the textural characteristics of foods with a high content of macromolecules such as proteins and starch. For instance, in meat and meat products, it has been used to modify their texture because of its capacity of improving the gelation behavior of myofibrillar protein systems. In 2013, Tokifuji et al. [18] applied high-pressure technology to grounded pork meat mixed with water in various proportions in order to obtain a soft pork meat gel with low hardness and elasticity and higher ease of swallowing. The authors reported that a gels to water ratio of 1:1 treated for 20 min at 400 MPa resulted in the highest softness, elasticity and smoothness and the lowest residue in the oropharynx, and suggested that HPP is a useful technology to create suitable dishes for people who suffer from dysphagia. Three years later, Yoshioka et al. [19] used the same technology and processing conditions to develop minced fish meat gel and confirmed that fish meat protein gel in meat to water ratios of 1:1 and 1:1.5 could be adequate for individuals who have difficulty with mastication and swallowing. It has also been suggested that HPP at ≥300 MPa could be an alternative to produce meat-based foods for dysphagia patients by reducing the hardness of foodstuffs [79].

HPP has also been successfully used for starchy [80] and puréed food products [81], resulting in low hardness and high viscosity, which can be claimed as adequate features for dysphagia diets. 

To our knowledge, after a careful review of the literature, no other technologies have been specifically applied to obtain TMF. Sungsinchai et al. [79], in a review that included potential technologies with possibilities to be applied to TMF designed for patients with dysphagia pointed out that the use of ultrasound, gamma irradiation, pulsed electric field treatment, plasma processing or high-hydrodynamic pressure processing might be good strategies to be explored in order to obtain suitable products for people with swallowing difficulties.

### 4.2. 3D Printing

Although textural properties are greatly important when dealing with the safety aspects in dysphagia, other sensory aspects such as color, shape, taste and odor should also be taken into account. 3D printing technology was developed by the additive manufacturing industry with great success, being basically a process of joining materials layer upon layer [82,83]. It can be a promising technology for the development of attractive dishes designed for dysphagia. Many food products have already been developed using 3D printing, but very few studies about dysphagia diet dishes using this technology have been published so far.

When 3D printing is used, several factors such as the raw material, the addition of hydrocolloids, the previous treatment process, the heat resistance, the shear-thinning degree of the sample and the ability to maintain the structural integrity after the deposition need to be considered in order to obtain the desired texture after storage, heating or cooking [20,84].

Kouzani et al. [85], reported that 3D printing reduced the design and fabrication time, improved consistency and repeatability of 3D-printed tuna fish elaborated with different ingredients (tuna, pureed beetroot and pumpkin) and improved the formulation. Moreover, Strother et al. [69] compared the effect of different hydrocolloids on the sensory and textural properties of 3D-printed carrots and molded carrots. The authors reported that printing did not change the sensorial aspects of the pureed carrots; therefore, it can be a good option to create visually and sensory-pleasing foods. In the same year, Dick et al. [20] studied the feasibility of 3D-printed pork elaborated with different hydrocolloids as dysphagia food. After comparing the impact on rheological, textural and microstructural properties of 3D-printed cooked paste with a various proportion of gums with the control sample, the author suggested that all the samples were suitable for their extrusion application and no significant rheological differences were observed among all the formulations when they were analyzed at room temperature. However, after freezing and heating, samples containing hydrocolloids showed lower hardness, cohesiveness and chewiness as compared to the control sample, as a result, those can be classified as transitional foods suitable for people with chewing and swallowing difficulties [20]. Recently, studies about the printability of modified texture of cooked beef pastes [53] and 3D printing of fresh vegetables using hydrocolloids have been reported [21].

The use of thickeners and non-thermal technologies in combination with 3D printing may help to develop safe, nutritional and sensory adequate foods for dysphagic patients. Table 3 summarizes some texture-modification technologies used in dysphagia management.

## 5. Conclusions

Patients affected by dysphagia are at higher risk of nutritional deficiencies and low survival rates. In order to increase the safe ingestion processes and adequate nutrition of these patients, the development of texture-modified foods is needed. In this context, TMF adapted for dysphagia patients such as pureed foods, milk formulas, vegetable creams, fruit juices or meat/fish patties have been optimized using empirical methods (LST, Bostwick consistometer, IDDSI) and also by measures of viscosity, viscoelasticity (G*, G′, G′′, yield stress), textural properties (hardness, cohesiveness, adhesiveness, gumminess) and tribological methods. Among the strategies to modulate these properties, the use of gums (xanthan and, more recently, gellan, tara, konjac or flaxseed) have shown some advantages as compared to the use of starches as thickeners, due to their better stability, solubility and viscosity properties. Finally, although not extensively studied yet, the application of high-pressure processing and 3D printing have evidenced promising results when developing foods for dysphagic patients.

## Figures and Tables

**Table 1:** Summary of the available methods and instruments that have been used in the last few years to characterize dysphagia foods.

Empirical methods	**Instruments**	**Parameters Measured**	**Characteristics**	**Applications**	**References**
	Line spread test	Viscosity	Measure the flow distance in centimeters traveled by the thickened fluid, across a flat surface, in 1 min	Commercial pureed foods	[50]
	Bostwick consistometer	Viscosity	Measure the flow distance in centimeters traveled by a thickened fluid, across a flat surface, in 30 s	Commercial pre-prepared thickened liquids	[51]
				Water	
				Juices (apple, cranberries, orange)	
	IDDSI syringe flow test	Viscosity	Measure the viscosity of dysphagia drinks by the gravity flow test (levels 0–4)	Commercial pre-prepared, thickened liquids	[51,52]
				Water	
				Juices (apple, cranberries, orange)	
	IDDSI spoon tilt test	Food cohesiveness	Determine the adhesiveness and cohesiveness of the sample (levels 4–5)	3D-printed fresh vegetables	[21]
		Adhesiveness			
	IDDSI fork pressure test spoon pressure test	Firmness	Determine the firmness of the sample	3D-printed pork paste	[20]
Fundamental methods	Rotational Shear Rheometer	Viscosity	Measure the viscosity of various thickened fluids at a wide range of shear rates by performing Steady Flow Sweep tests	Water	[33,35,36]
				Fruit juices	
				Milk	
				Pureed foods	
		Viscoelasticity (Complex modulus G*, tan δ and Yield stress)	Measured by Oscillation Frequency Sweep tests and Oscillation Amplitude Sweep tests	Commercial thickeners	[35,36,39]
				Pureed foods	
	Extensional rheometer	Extensional flow	The maximum extensional viscosity is related to the cohesiveness of the fluid	Commercial thickeners	[15]
	Tribometer	Slipperiness	Measures the degree of lubrication	Commercial thickeners mixed with water, milk and orange	[13]
	Texture analyzer	Hardness	Measures the mechanical properties of pureed foods and texture-modified foods by performing back-extrusion tests in pureed foods and TPA tests in texture-modified foods	Various thickened pureed foods for dysphagia patients: chickpea stew, chicken stew, lentils with rice, halibut with green sauce, pasta bolognese, beef pastes	[23,53]
		Cohesiveness			
		Adhesiveness			
		Gumminess			

**Table 2:** Summary of textural and technological properties of some starch-based and gum-based thickeners used in the last few years in dysphagia management.

Thickener Type	General Properties	Thickeners	Uses	Characteristics	References
Starch-based	Consistency alters over time	Corn starch	Pureed carrots	High adhesiveness; therefore, a bolus difficult to swallow	[36]
	Susceptible to hydrolysis	Tapioca starch	Distilled water	Good thickening agent for instant consumption due to its solubility	[26]
	Increased prevalence of pharyngeal residue		Sport drinks		
	Grainy texture		Orange juice		
	Cloudy appearance				
Gum-based	Stable over the time Amylase-resistant Temperature and pH stability Low oropharyngeal residue Soft uniform texture Clear appearance Tasteless Odorless	Xanthan gum	Fruit juices Milk Water Pork paste Pureed vegetables	Amylase-resistant	[21,33,36,42,54,58,59]
				Temperature and pH stability	
				Low oropharyngeal residue	
				Clear appearance	
				Tasteless	
				Odorless	
				Shear-thinning behavior	
				The banana gel containing agar was considered suitable for the elderly	
		Agar	Banana dessert gels	Capacity to form soft gels	[37]
		Carboxymethyl cellulose	Tailor-made thickened pea cream	Therapeutic properties: prevent the occurrence of colorectal cancer, promoting an improvement in postprandial glycemia and weight control Presents phenolic compounds that could exhibit pharmacological properties including antidiabetic, antihypertensive, immunomodulatory, anti-inflammatory and neuro-protective properties.	[35]
		Flaxseed gum	Water Milk Orange-flavored soy juice	Good lubrication profile α-amylase resistance	[13,60]
		Gellan gum	Water Pureed carrots	Provide a suitable texture for people with chewing and swallowing difficulties	[36,61]
		Guar gum	Pork paste	Provide a good viscous component and a bolus easier to swallow	[20]
		Konjac gum	Tailor-made thickened pea cream	Provide a good viscous component and a bolus easier to swallow	[35]
		Tara gum	Tailor-made thickened pea cream		[35]

**Table 3:** Texture modification technologies used in dysphagia management.

Technology	Characteristics	Food Product Applications	Suggested Processing Conditions	Results	References
High-pressure processing (HPP)	Improve the gelation behavior of protein by modifying its conformational structure	Pork meat gel	Pressurization parameters: 400 MPa for 20 min at 17 ± 2 °C	Gel to meat/water ratio of 1:1 were those with the lowest residue in the oropharynx and highest smoothness, softness and elasticity and could be used to create dishes for a dysphagia diet	[18,79,86]
	Retain flavors and nutrients Improve bioavailability of bioactive compounds	Minced fish meat gel	Pressurization parameters: 400 MPa for 20 min at 17 ± 2 °C	Gel to meat/water ratios of 1:1 and 1:1.5 were slightly elastic and smooth and, in consequence, suitable for a dysphagia diet	[19]
3D Printing	3D printing is a potential alternative technique for the elaboration of sensorial attractive dishes and contribution to the formulation of innovative food products	Tuna fish, molded carrots, pork paste, fresh vegetables, beef paste	The texture of the feeding material should be adapted to the 3D-printing processing:	3D printing is an emerging technology that can be used to create attractive dishes that maintain the shape of foods and their sensory and textural properties The method reduced the time and cost of design and fabrication, reduced the reliance on a skilled cook and improved the visual appearance, consistency and repeatability of the foods produced	[20,21,53,69,85]
			Preparation of the sample (material, formulation, amount of thickener		
			Ensure the shear thinning behavior		
			Nozzle size		
			Layer height		
			Layer height		
			Layer height		
			Print speed		
			Structure maintenance		

## Data Availability

Not applicable.
